# Median effective dose and 95% effective dose of oliceridine combined with ciprofol for painless gastroscopy: a dose-finding study based on Dixon’s up-and-down method

**DOI:** 10.3389/fphar.2026.1918438

**Published:** 2026-09-04

**Authors:** Xikun Sun, Pei Song, Bin Zou, Yimeng Zhuang, Xiaohang Han, Jianyu Zu, Yugang Diao

**Affiliations:** 1 Department of Anesthesiology, General Hospital of Northern Theater Command, Shenyang, Liaoning, China; 2 Department of Anesthesiology, Suzhou BOE Hospital, Suzhou, Jiangsu, China

**Keywords:** ciprofol, ED50, ED95, oliceridine, painless gastroscopy

## Abstract

**Objective:**

To determine the median effective dose and estimate 95% effective dose of oliceridine combined with ciprofol for suppressing gastroscope insertion responses, stratified by sex.

**Methods:**

This prospective, sex-stratified trial enrolled patients between 16 January and 31 March 2026, at the General Hospital of Northern Theater Command. Patients, stratified into separate male and female cohorts, received an individualized dose of oliceridine followed by a fixed dose of ciprofol (0.4 mg/kg) for anesthesia induction. The oliceridine dose was determined using a modified Dixon up-and-down method, with an initial dose of 0.02 mg/kg. Subsequent doses increased or decreased by a ratio of 1.0:1.1 based on positive or negative responses. The positive response was defined as retching, coughing, body movement, or a greater than 30% increase in mean arterial pressure or heart rate within 1 min of insertion. The trial terminated upon reaching seven consecutive response reversals in each cohort.

**Results:**

A total of 29 male and 32 female patients completed the study. The ED_50_ and ED_95_ values of oliceridine were 12.74 μg/kg (95% CI: 9.48–14.76) and 17.69 μg/kg (95% CI: 15.32–40.14) in females, compared with 17.37 μg/kg (95% CI: 15.59–19.01) and 20.98 μg/kg (95% CI: 19.22–32.47) in males, respectively. In both the male and female cohorts, ciprofol consumption was significantly lower in the negative-response group than in the positive-response group, whereas oliceridine requirements were higher. At gastroscope insertion, both MAP and HR decreased, with the negative-response group showing significantly lower values. SpO_2_ levels at insertion were significantly higher than baseline values. There were no significant differences in the incidence of adverse events between the two sexes.

**Conclusion:**

For day-case painless gastroscopy, the ED_50_ of oliceridine combined with ciprofol (0.4 mg/kg) was 12.74 μg/kg in females and 17.37 μg/kg in males, with ED_95_ estimates of 17.69 μg/kg and 20.98 μg/kg, respectively.

## 1 Background

Gastroscopy is the standard modality for upper gastrointestinal diseases, providing the morphological detail to detect erosions, ulcers, and early neoplasms (Dinis-Ribeiro and Areia, 2025). Nevertheless, the procedure frequently provokes intense defensive reflexes, including retching, a sensation of laryngeal spasm, hypersalivation, and abdominal bloating. Combined with preoperative anxiety, these reactions represent the primary sources of psychological distress (Nguyen et al., 2024; Neilson and Rees, 2025). A clinical study evaluating patients undergoing sedated endoscopy identified younger age (<40 years), female sex, high body mass index (≥25 kg/m^2^) and hiatal hernia as independent risk factors for poor intraprocedural cooperation, manifested primarily as coughing and somatic movement (Lee et al., 2017). Another prospective trial identified younger age, high trait anxiety, and pharyngeal topical anesthesia as independent predictors of poor tolerance (Campo et al., 2000). The physiological and psychological burdens not only compromise patient compliance with follow-up appointments but also disrupt visual stability owing to body movements, potentially resulting in the oversight of early minor lesions during diagnosis (Kanzaki et al., 2024). Driven by the integration of comfort-directed healthcare, painless gastrointestinal endoscopy has gradually emerged as the preferred modality for patients. The American Society for Gastrointestinal Endoscopyguidelines recommend a combination of opioids and propofol for sedation and anesthesia during endoscopic procedures (Early et al., 2018). Oliceridine, a novel G-protein biased µ-opioid receptor agonist, provides effective analgesia while minimizing β-arrestin-2-mediated respiratory depression and opioid-related adverse events (Daksla et al., 2023; Wolf et al., 2024). Clinical evidence demonstrates that the co-administration of oliceridine and propofol for gastroscopy reduces the incidence of respiratory depression, postoperative nausea and vomiting without prolonging recovery time (Ma et al., 2025). Complementarily, ciprofol selectively modulates GABAA receptors to induce rapid hypnotic depth, offering a lower incidence of respiratory depression and injection pain, alongside superior patient satisfaction compared to propofol. (Ortegal et al., 2024). Mechanistically, combining these two agents engages a dual-target synergy that yields a potent dose-sparing effect, maintaining optimal sedation and analgesia without compounding cardiorespiratory depression. In summary, while both oliceridine and ciprofol show favorable clinical profiles in sedated gastroscopy, data regarding their co-administration remain unavailable. Additionally, the influence of sex on opioid analgesic response remains to be fully elucidated. This study primarily aimed to determine the ED_50_ of oliceridine combined with ciprofol for painless gastroscopy, with the ED_95_ evaluated as an exploratory endpoint, stratified by sex, to provide an evidence-based reference for rational clinical medication.

## 2 Materials and methods

### 2.1 Study design

The study received approval from the Institutional Review Board of the General Hospital of Northern Theater Command and was registered with the Chinese Clinical Trial Registry (ChiCTR2600116676). All procedures were conducted in accordance with the Declaration of Helsinki, and informed consent was obtained from every participant.

### 2.2 Participants

This study enrolled patients scheduled for elective painless gastroscopy between 16 January and 31 March 2026. Inclusion criteria: aged 18–60 years, BMI 18.5–28.0 kg/m^2^, ASA class I or II. Exclusion criteria included: allergy or contraindication to the study medication, upper gastrointestinal bleeding or significant abnormalities in coagulation function, severe dysfunction of vital organs, modified Mallampati grade ≥3, long-term history of psychotropic drug use, or cognitive dysfunction unable to cooperate. Exclusion criteria include the requirement for endoscopic therapy or withdrawal from the study for any reason.

### 2.3 Perioperative anesthetic care

All patients were routinely fasted for 8 h and restricted fluid intake for 2 h before surgery. Upon arrival in the operating room, peripheral venous access was established and standard monitoring was initiated, including noninvasive blood pressure, electrocardiogram, and peripheral oxygen saturation (SpO_2_). Prior to induction of anesthesia, patients underwent preoxygenation via a nasal cannula at 3 L/min for 3 min, and oxygen administration via the nasal cannula was maintained intraoperatively. All procedures were performed by the same team of surgeons, anesthesiologists, and nurses. During anesthesia induction, oliceridine was first administered as a slow intravenous bolus over 30 s, followed 3 min later by intravenous ciprofol (0.4 mg/kg) over 30 s. Gastroscopy was initiated approximately 6 min after oliceridine administration and 2 min following ciprofol injection, when the Modified Observer’s Assessment of Alertness/Sedation (MOAA/S) score decreased to one or less. Hypoxemia (SpO_2_ < 90%) was managed with a jaw-thrust maneuver or bag-mask ventilation as required. Hypotension, defined as a mean arterial pressure (MAP) decrease of greater than 20% from baseline or MAP <65 mmHg, was treated with ephedrine 5 mg intravenously. Bradycardia, heart rate (HR) < 50 beats/min, was corrected with intravenous atropine 0.5 mg.

### 2.4 The modified Dixon’s up-and-down sequential allocation method

The individual dose of oliceridine was determined using a modified up-and-down sequential allocation method. Based on prior investigations, the initial dose was set at 0.02 mg/kg, with adjacent dose levels adjusted by a fixed ratio of 1.0:1.1. A positive response was defined as the occurrence of coughing, retching, body movement, or a greater than 30% increase from baseline in HR or MAP during or within 1 min following gastroscope insertion; otherwise, the response was classified as negative. If a positive response disrupted the procedure, a rescue bolus of ciprofol 10 mg was administered. For persistent or inadequate control, supplemental alfentanil 5 μg/kg was delivered. A shift from positive and negative responses was defined as a reversal, and the trial was terminated upon the completion of seven consecutive reversals.

### 2.5 Outcomes

#### 2.5.1 Primary outcomes

The ED_50_ with 95% CI of oliceridine combined with ciprofol for suppressing gastroscope insertion responses, stratified by sex.

#### 2.5.2 Secondary outcomes

The ED_95_ was assessed as a secondary exploratory outcome.

### 2.6 Perioperative safety and procedural monitoring

HR, MAP, and SpO_2_ were measured at the following time points: upon operating room admission (T1), gastroscope insertion (T2), gastroscope withdrawal (T3), and prior to discharge (T4). Baseline characteristics, including age, BMI, and ASA physical status, along with procedural duration and total ciprofol consumption, were recorded. Perioperative adverse events were recorded, including injection pain, hypoxemia (SpO_2_ ≤ 90%), hypotension, bradycardia, dizziness, nausea, vomiting, and fatigue.

### 2.7 Statistical analysis

Statistical analyses were performed using SPSS version 25.0 (IBM Corp., Armonk, NY, United States). Normally distributed continuous variables are expressed as mean ± standard deviation (SD), and intergroup comparisons were conducted using independent-samples t-tests. Non-normally distributed continuous variables were expressed as medians (interquartile ranges) and compared by rank-sum tests. Categorical variables were expressed as frequencies (percentages) and analyzed using Pearson’s chi-square test or Fisher’s exact test. Drug doses administered via the geometric sequence (1:1.1 ratio) were logarithmically transformed (log1_0)_ to obtain an arithmetic dose scale for analysis. Probit regression analysis was then performed to calculate the ED_50_ and ED_95_ of oliceridine (with 95% CIs), which were subsequently anti-log-transformed back to linear dose units (µg/kg). The up-and-down sequence plot and the dose–response curve were generated by GraphPad Prism9.0.0 (GraphPad Software Inc., San Diego, CA, United States). Normally distributed repeated-measures data were analyzed using repeated-measures ANOVA (RM-ANOVA), whereas non-normally distributed data were analyzed using generalized estimating equations (GEE). If a significant group-by-time interaction effect was detected, *post hoc* between-group simple effect analyses were performed. A two-tailed P value <0.05 was considered statistically significant. To adjust for multiple comparisons across the four time points, a Bonferroni-corrected significance threshold of α = 0.0125 (0.05/4) was applied for *post hoc* pairwise comparisons.

## 3 Results

**FIGURE 1:**
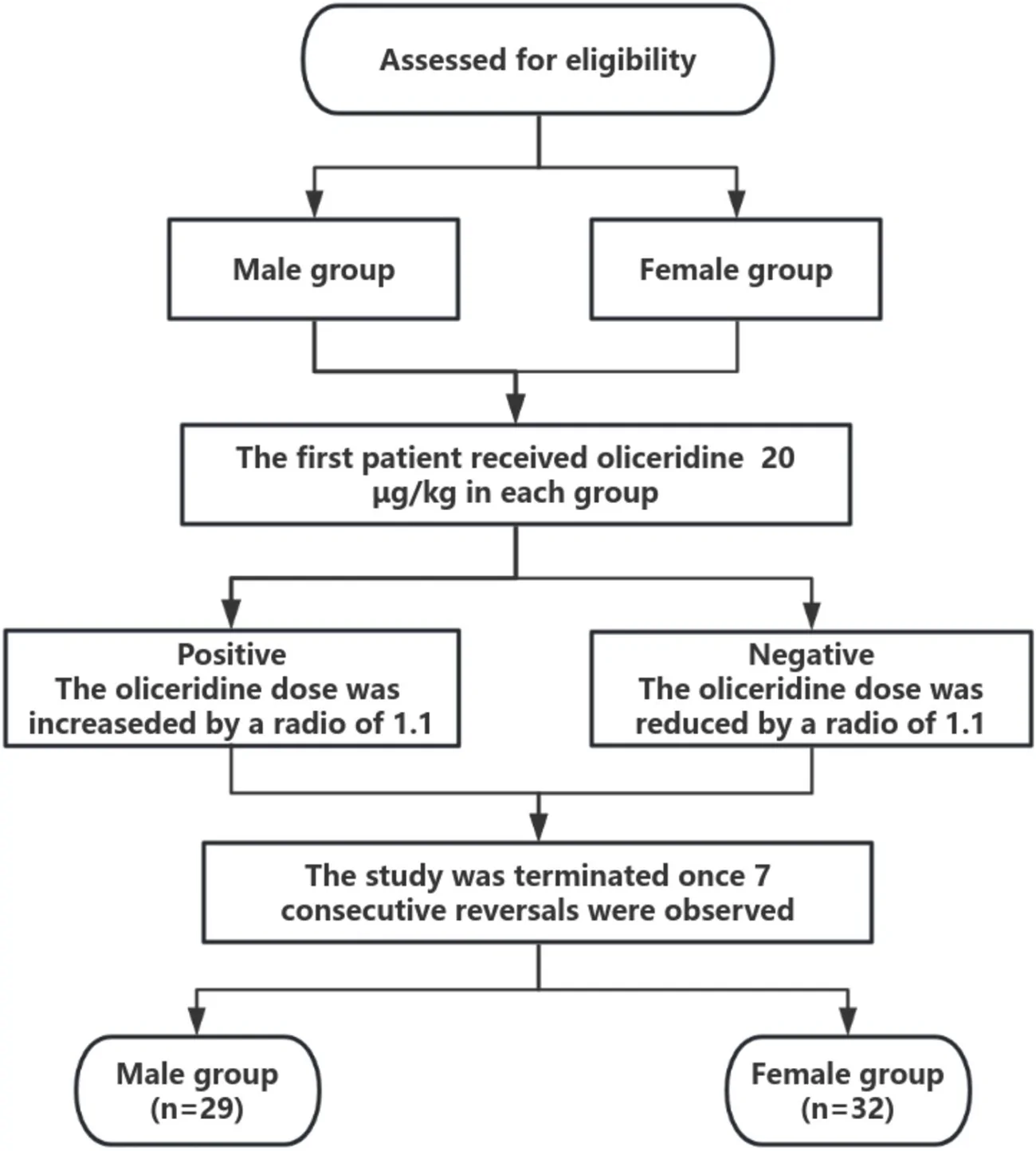
The flowchart of the study.

A total of 29 male and 32 female patients were enrolled, with no withdrawals or loss to follow-up (Figure 1). With stratification by sex, baseline characteristics, including age, BMI, ASA physical status classification, and gastroscopy duration, showed no statistical differences between the positive-response and negative-response groups. In both male and female cohorts, ciprofol consumption was significantly lower in the negative-response group than in the positive-response group, whereas oliceridine doses were significantly higher in the negative-response group (Table 1).

**FIGURE 2:**
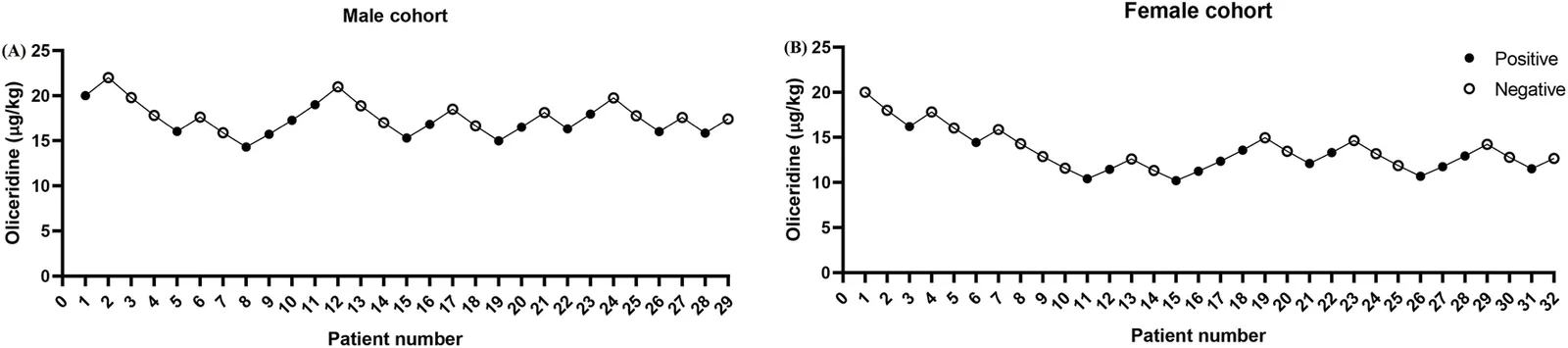
Individual response to gastroscopic insertion stimulus in male **(A)** and female **(B)** cohorts. Solid circles and open circles represent positive and negative responses, respectively.

**FIGURE 3:**
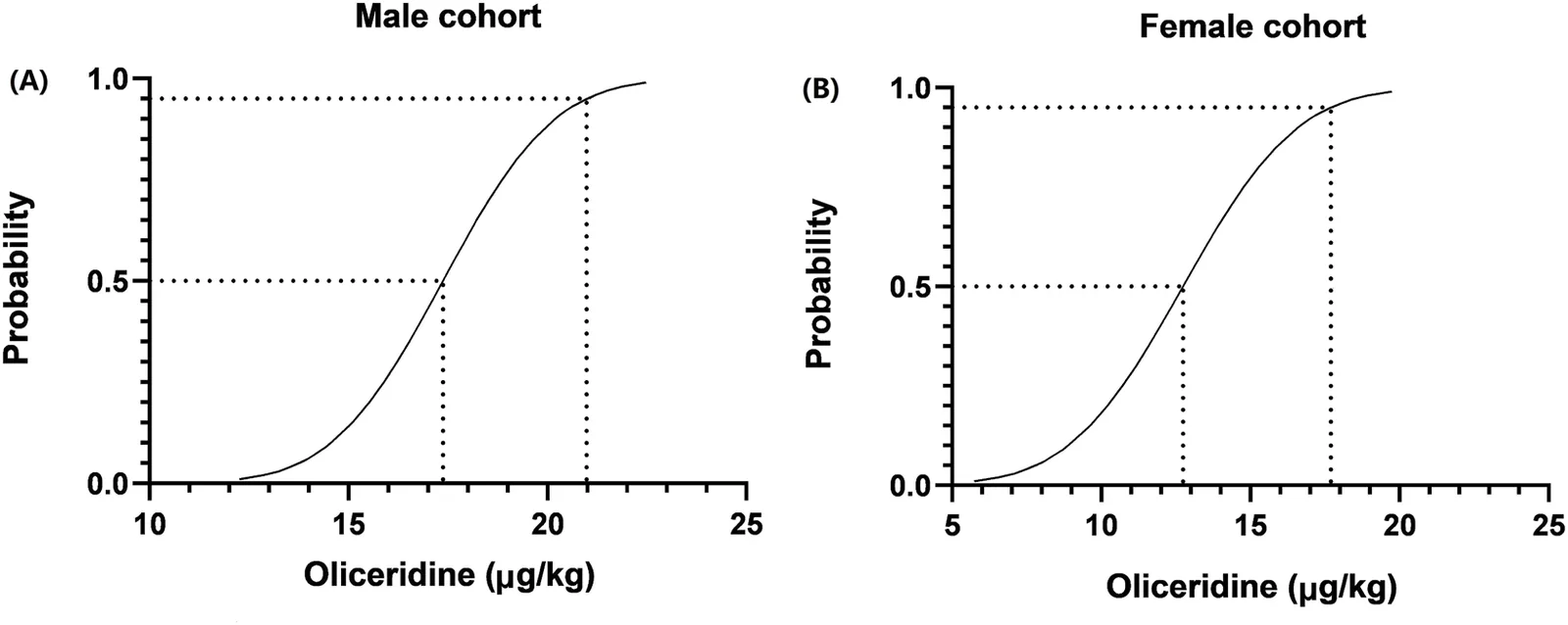
Dose-response curve of oliceridine in painless gastroscopy for male **(A)** and female **(B)** cohorts.

The trial was terminated upon reaching seven consecutive response reversals. The sequential allocation of positive and negative responses to oliceridine fumarate during painless gastroscopy was illustrated in Figure 2. The ED_50_ and ED_95_ values of oliceridine combined with ciprofol in painless gastroscopy were 12.74 μg/kg (95% CI: 9.48–14.76) and 17.69 μg/kg (95% CI: 15.32–40.14) in the female group, and 17.37 μg/kg (95% CI: 15.59–19.01) and 20.98 μg/kg (95% CI: 19.22–32.47) in the male group, respectively (Figure 3).

Repeated-measures ANOVA revealed significant group and time main effects for MAP in both male and female cohorts (*P* < 0.001). Crucially, significant group-by-time interaction effects were observed in both sexes (males: F = 5.598, *P* = 0.004; females: F = 6.638, *P* < 0.001), indicating that the longitudinal trajectories of MAP differed inherently based on the procedural response. Post-hoc pairwise comparisons for the time main effect revealed that, in both male and female cohorts, mean MAP values at T2, T3, and T4 were all significantly lower than at T1 (*P* < 0.001), with all differences remaining significant after Bonferroni correction. Analysis of the group main effect demonstrated that mean MAP across all time points was significantly higher in positive responders than in negative responders (P < 0.001). At T1, MAP was comparable between the positive- and negative-response groups across both sexes. However, at T2, MAP decreased from baseline in all patients, with the analysis revealing a significantly lower MAP in the negative-response group compared with the positive-response group in females (*P* = 0.002). A similar lower trend was observed in males at T2 (*P* = 0.017), though it did not reach statistical significance after the Bonferroni *post hoc* adjustment. At T3 and T4, no statistically significant intergroup differences were detected in either sex.

For HR, main effects of group and time were significant in both sexes (F = 13.320, *P* < 0.001). A significant group-by-time interaction occurred in males (*P* < 0.001). Post-hoc pairwise comparisons for the time main effect showed that mean HR values at T2, T3, and T4 were significantly lower than at T1 in both sexes (*P* < 0.001), all retaining statistical significance after Bonferroni adjustment. Furthermore, the main effect of group indicated that overall HR was significantly higher in the positive-response group relative to the negative-response group (*P* < 0.001). In the male cohort, the analysis demonstrated a significantly greater reduction from T1 to T2 in the negative-response group compared with the positive-response group (*P* < 0.001). The difference retained statistical significance following *post hoc* Bonferroni correction. At T2, compared to the negative-response group, higher HR values observed in the positive-response group failed to reach statistical significance after *post hoc* Bonferroni adjustment in either sex. In contrast, no significant group-by-time interaction was observed in females (F = 1.127, *P* = 0.337), and thus simple effect analyses were not indicated.

GEE demonstrated that SpO_2_ was significantly influenced by the main effects of group (Waldχ^2^ = 4.244, *P* = 0.039; females: Waldχ^2^ = 3.231, *P* = 0.045) and time (*P* < 0.001). However, no significant group-by-time interactions were detected in either cohort (males: *P* = 0.062; females: *P* = 0.072). The significant group main effect indicated that, the mean SpO_2_ was higher in the negative-response group than in the positive-response group in both sexes. Post-hoc pairwise comparisons for the time main effect with Bonferroni adjustment showed that SpO_2_ was significantly higher at T2 and T3 than at T1 in both male and female cohorts (*P* < 0.001). Because group-by-time interactions were not statistically significant, *post hoc* simple effect testing across individual time points was omitted (Table 2 and Supplementary Table S1).

**TABLE 1:** Baseline characteristics.

Variables	Male (n = 29)		*P*	Female (n = 32)		*P*
	Positive (n = 14)	Negative (n = 15)		Positive (n = 14)	Negative (n = 18)	
Age (years)	37.00 ± 6.86	39.73 ± 7.03	0.299	37.93 ± 8.86	39.83 ± 10.86	0.598
BMI(kg/m^2^)	24.19 ± 2.23	24.78 ± 2.71	0.524	22.86 ± 2.93	23.42 ± 2.30	0.544
ASA I/II	4/10	6/9	0.700	5/9	8/10	0.725
Procedure duration(s)	195.00 (185.75, 232.00)	211.00 (191.50, 220.25)	0.285	206.00 (191.75, 219.50)	198.00 (187.50, 246.75)	0.866
Ciprofol dosage (mg)	41.21 ± 3.85	32.47 ± 4.64	<0.001	35.36 ± 3.32	26.67 ± 2.66	<0.001
Oliceridine dosage (ug)	1,174.86 (1,125.81, 1325.38)	1,319.02 (1,157.65, 1689.43)	0.015	721.43 (661.20, 788.01)	830.78 (732.82, 991.94)	0.010

**TABLE 2:** Comparison of perioperative hemodynamics and oxygenation status at different times.

Parameters	Male (n = 29)		*P*	Female (n = 32)		*P*
	Positive (n = 14)	Negative (n = 15)		Positive (n = 14)	Negative (n = 18)	
MAP(mmHg)						
T1	100.50 ± 7.06	101.47 ± 7.41	0.722	101.14 ± 10.78	98.67 ± 11.90	0.548
T2	92.57 ± 7.45	85.20 ± 8.06	0.017	93.57 ± 7.40	83.72 ± 8.38	0.002
T3	81.64 ± 7.79	84.60 ± 7.36	0.388	78.50 ± 6.99	82.17 ± 10.16	0.258
T4	91.07 ± 7.40	94.20 ± 8.15	0.290	89.64 ± 7.14	90.94 ± 7.66	0.672
HR(bmp)						
T1	81.14 ± 11.66	82.53 ± 9.78	0.766	87.64 ± 9.95	87.17 ± 10.24	–
T2	75.79 ± 13.84	66.40 ± 8.51	0.035	81.43 ± 9.22	74.50 ± 9.64	–
T3	71.29 ± 9.66	68.73 ± 10.12	0.494	71.79 ± 8.92	73.50 ± 9.24	–
T4	74.86 ± 8.01	73.80 ± 7.73	0.673	78.71 ± 5.97	77.78 ± 7.16	–
SpO_2_ (%)						
T1	97.00 (97.00, 98.00)	98.00 (97.00, 99.00)	–	97.00 (96.75, 99.00)	97.00 (97.00, 98.00)	–
T2	99.00 (98.00, 100.00)	99.00 (98.00, 99.00)	–	98.00 (98.00, 99.00)	98.00 (98.00, 100.00)	–
T3	98.00 (98.00, 99.00)	99.00 (98.00, 100.00)	–	98.00 (98.00, 99.00)	98.00 (97.75, 99.00)	–
T4	97.00 (96.75, 98.00)	98.00 (97.00, 99.00)	–	97.50 (97.00, 98.00)	98.00 (97.00, 99.00)	–

T1: upon operating room admission; T2: gastroscope insertion; T3: gastroscope withdrawal; T4: prior to discharge. –: Post-hoc pairwise comparisons between groups at individual time points were not conducted because the group-by-time interaction effect was not statistically significant.

**TABLE 3:** Comparison of perioperative adverse events.

Adverse events	Male (n = 29)	Female (n = 32)	*P*
Hypotension, n (%)	2 (6.90%)	1 (3.13%)	0.600
Dizziness, n (%)	2 (6.90%)	1 (3.13%)	0.600
Nausea, n (%)	1 (3.45%)	3 (9.38%)	0.614
Fatigue, n (%)	0 (0%)	1 (3.13%)	1.000
Bradycardia, n (%)	2 (6.90%)	2 (6.25%)	1.000
Vomiting, n (%)	0 (0%)	0 (0%)	—
Injection pain, n (%)	0 (0%)	0 (0%)	—

No injection pain or hypoxemia occurred in either group. In the male cohort, adverse events included hypotension (n = 2), bradycardia (n = 2), and nausea (n = 1); no instances of vomiting, dizziness, or fatigue were reported. In the female cohort, adverse events included nausea (n = 3), bradycardia (n = 2), hypotension (n = 1), dizziness (n = 1), and fatigue (n = 1), with no vomiting observed (Table 3).

## 4 Discussion

This study first determined the dose-response parameters of oliceridine combined with ciprofol in inhibiting the insertion response during painless gastrointestinal endoscopy via a modified Dixon up-and-down method. By probit regression analysis, the ED_50_ and ED_95_ values of oliceridine to suppress the endoscope insertion response were 12.74 μg/kg (95% CI: 9.48–14.76) and 17.69 μg/kg (95% CI: 15.32–40.14)in the female group, compared with 17.37 μg/kg (95%CI: 15.59–19.01) and 20.98 μg/kg (95% CI: 19.22–32.47) in the male group, respectively.

Cao et al. reported that ED_50_ and ED_95_ values of oliceridine combined with propofol for inhibiting responses to painless gastrointestinal endoscope insertion were 12.63 μg/kg (95% CI: 11.43–13.79) and 14.46 μg/kg (95% CI: 13.41–20.33) in males, and 10.38 μg/kg (95% CI: 9.02–11.96) and 13.19 μg/kg (95% CI: 11.62–28.23) in females (Cao et al., 2025). Meanwhile, Tang et al. found that the ED_50_ and ED_95_ values of oliceridine were 22.5 and 23.8 μg/kg, respectively (Tang et al., 2025). These divergences from our current findings may be closely associated with the distinct molecular receptor mechanisms of different sedative agents as well as the variations in research methodologies. The affinity of ciprofol for GABA_a_ receptors is 4–5 times greater than that of propofol (Zhou et al., 2025). This difference in binding characteristics may distinguish the pharmacodynamic interactions of ciprofol combined with μ-opioid receptor agonists from those of propofol. Propofol exerts allosteric activation or potentiation at multiple GABA_a_ receptor subunit interfaces, including the β-β, α-β, and β-α interfaces (Eaton et al., 2016), which may mediate different downstream effects. Although ciprofol also targets the GABAA receptor, the introduction of its cyclopropyl side chain reconfigures the molecule’s steric effects and lipophilicity (Zhou et al., 2025). This alteration may affect its binding modes at various subunit interfaces, leading to a different dose-response relationship when combined with opioids.

Sex differences exist in the analgesic effects and adverse events of opioids (Nasser and Afify, 2019). Consistent with previous finding (Cao et al., 2025), the ED_50_ and ED_95_ values in the present study were significantly higher in males than in females. A review by Fillingim et al. indicated that females exhibit greater analgesic responses to μ-opioid receptor agonists in human experimental pain models (Fillingim and Gear, 2004). Additionally, neuroimaging evidence has demonstrated higher μ-opioid receptor binding capacity in cortical and subcortical regions in females (Bodnar and Kest, 2010). These findings were further supported by a systematic review and meta-analysis of 50 studies, which showed that females achieve significantly better analgesic efficacy than males in patient-controlled analgesia studies, with this sex difference becoming more pronounced with extended PCA duration (Niesters et al., 2010). The modulation of opioid receptors by gonadal hormones may also play a key role in this process, with this interaction enhancing the inhibitory effects of opioids on pain responses in females (Joe et al., 2016). Pharmacokinetically, females typically exhibit a higher body fat percentage and an expanded volume of distribution for lipophilic opioids. Coupled with sex-specific differences in hepatic clearance, these factors may prolong plasma elimination and increase effect-site drug exposure (Pleym et al., 2003). Furthermore, at the circuit level, dimorphic expression of endogenous opioid peptides and differential functional connectivity within the periaqueductal gray-rostral ventromedial medulla descending pain-inhibitory pathway may further sensitize females to visceral analgesia (Loyd and Murphy, 2009; Mogil, 2012; Sorge and Totsch, 2017). Consequently, the findings underscore that clinicians should consider sex as an important factor in personalized opioid dosing, alongside age, weight, and comorbidities (Lau et al., 2021). Translationally, defining sex-stratified ED_50_ and ED_95_ values provides distinct clinical utility for individualized sedation (Muller and Milton, 2012). The ED_50_ metrics (12.74 μg/kg in females vs. 17.37 μg/kg in males) quantify underlying pharmacodynamic sensitivity, whereas the corresponding ED_95_ values (17.69 μg/kg in females vs. 20.98 μg/kg in males) serve as clinical dosing targets to prevent insertion responses in over 95% of patients. Applying these sex-specific thresholds enables precise drug titration, reducing the risk of relative over-sedation in females and inadequate antinociception in males during day-case procedures.

In the present study, the combination of oliceridine and ciprofol demonstrated a favorable safety profile. The adverse events in the male cohort were limited to hypotension (n = 2) and nausea (n = 1). Similarly, the female cohort experienced nausea (n = 3), hypotension (n = 2), dizziness (n = 1), and fatigue (n = 1). Hypoxemia was not observed in the study, which can be partly attributed to the G-protein-biased agonism profile of oliceridine. By selectively activating the G-protein pathway and reducing β-arrestin recruitment, oliceridine maintains a minimal risk of respiratory depression while achieving adequate analgesia. Consistent with previous literature (Ma et al., 2025), the overall incidence of postoperative nausea and vomiting remained low in the study. Nevertheless, the incidence of nausea was notably higher in females than in males (9.38% vs. 3.45%). This difference is likely multifactorial. Female is an independent risk factor for postoperative nausea and vomiting (Lopes et al., 2021), and fluctuations in estrogen levels may modulate 5HT3 receptor sensitivity, thereby lowering the emetic threshold (Roche and King, 2015). Furthermore, no incidence of injection pain was reported which may be attributed to the milder chemical irritation of intravascular nerve endings by ciprofol at equivalent sedation depths, as well as its lower total intravascular drug exposure (Lu et al., 2023).

This study has several limitations. First, although Dixon’s up-and-down method is the standard approach for ED_50_ estimation, it presents inherent methodological limitations when extrapolating high-percentile doses such as ED_95_. Because data collection is concentrated around the median effect level, Probit regression analysis inevitably yields wider 95% confidence intervals at the distribution tails—an effect particularly pronounced in our female cohort (17.69 μg/kg, 95% CI: 15.32–40.14 μg/kg). This marked dispersion reflects reduced statistical precision and potential pharmacodynamic heterogeneity among female subjects rather than a definitive dosing range. Consequently, the calculated ED_95_ values should be interpreted strictly as exploratory estimates. Future studies aiming to establish precise high-percentile dosing targets should utilize specialized designs, such as the Biased Coin Design. Second, due to clinical constraints, respiratory assessment was based solely on pulse oximetry SpO_2_, without more sensitive monitoring such as end-tidal carbon dioxide, EtCO_2_ partial pressure. Supplemental oxygen can mask desaturation during hypoventilation, SpO_2_ alone may fail to detect early respiratory depression. Subsequent studies should incorporate, EtCO_2_ monitoring to more comprehensively evaluate the risk of respiratory depression. Third, our findings have limited generalizability due to the single center design, relatively small sample size, and restriction to ASA I–II patients, leaving the efficacy in higher-risk or elderly populations (ASA III–IV) unverified. Additionally, the lack of real time PK/PD plasma sampling and recovery quality assessment warrants future multicenter trials incorporating formal PK/PD modeling and postprocedural recovery metrics.

## 5 Conclusion

The ED_50_ and ED_95_ values of oliceridine combined with ciprofol for inhibiting responses to gastroscopic insertion were 12.74 μg/kg (95% CI: 9.48–14.76) and 17.69 μg/kg (95% CI: 15.32–40.14) in females, and 17.37 μg/kg (95% CI: 15.59–19.01) and 20.98 μg/kg (95% CI: 19.22–32.47) in males, respectively. No hypoxemia or injection pain occurred, while other adverse events were mild and uncommon.

## Data Availability

The raw data supporting the conclusions of this article will be made available by the authors, without undue reservation.

## References

[B1] Bodnar R. J. Kest B. (2010). Sex differences in opioid analgesia, hyperalgesia, tolerance and withdrawal: central mechanisms of action and roles of gonadal hormones. Horm. Behav. 58 (1), 72–81. 10.1016/j.yhbeh.2009.09.012 19786031

[B2] Campo R. Brullet E. Montserrat A. Calvet X. Donoso L. Bordas J. M. (2000). Efficacy of low and standard midazolam doses for gastroscopy. A randomized, double-blind study. Eur. J. Gastroenterol. Hepatol. 12 (2), 187–190. 10.1097/00042737-200012020-00009 10741933

[B3] Cao J. Gu X. Zhang X. Cheng Y. Jiang L. (2025). Determination of the effective dose of oliceridine combined with propofol using the modified Dixon's up-and-down method in painless gastroscopy. Front. Pharmacol. 16, 1620158. 10.3389/fphar.2025.1620158 41221038 PMC12597933

[B4] Daksla N. Wang A. Jin Z. Gupta A. Bergese S. D. (2023). Oliceridine for the management of moderate to severe acute postoperative pain: a narrative review. Drug Design Develop. Therapy 17, 875–886. 10.2147/DDDT.S372612 36987403 PMC10040154

[B5] Dinis-Ribeiro M. Areia M. (2025). How to improve the quality of upper gastrointestinal diagnostic endoscopy? Clin. Endosc. 58 (5), 633–637. 10.5946/ce.2024.339 40195643 PMC12489563

[B6] Early D. S. Lightdale J. R. Vargo J. J. N. Acosta R. D. Chandrasekhara V. Chathadi K. V. (2018). Guidelines for sedation and anesthesia in GI endoscopy. Gastrointest. Endosc. 87 (2), 327–337. 10.1016/j.gie.2017.07.018 29306520

[B7] Eaton M. M. Germann A. L. Arora R. Cao L. Q. Gao X. Shin D. J. (2016). Multiple non-equivalent interfaces mediate direct activation of GABAA receptors by propofol. Curr. Neuropharmacol. 14 (7), 772–780. 10.2174/1570159x14666160202121319 26830963 PMC5050400

[B8] Fillingim R. B. Gear R. W. (2004). Sex differences in opioid analgesia: clinical and experimental findings. Eur. J. Pain (London, England) 8 (5), 413–425. 10.1016/j.ejpain.2004.01.007 15324773

[B9] Joe H. B. Kim J. Y. Kwak H. J. Oh S. E. Lee S. Y. Park S. Y. (2016). Effect of sex differences in remifentanil requirements for the insertion of a laryngeal mask airway during propofol anesthesia: a prospective randomized trial. Med. (Baltimore) 95 (39), e5032. 10.1097/MD.0000000000005032 27684878 PMC5265971

[B10] Kanzaki H. Kuraoka S. Satomi T. Okanoue S. Hamada K. Kono Y. (2024). Analysis of painful situations during unsedated esophagogastroduodenoscopy. Endosc. Int. Open. 12 (11), E1267–E1276. 10.1055/a-2401-6804 39524195 PMC11543283

[B11] Lau T. Hayward J. Vatanpour S. Innes G. (2021). Sex-related differences in opioid administration in the emergency department: a population-based study. Emerg. Med. J. 38 (6), 467–473. 10.1136/emermed-2020-210215 33853938

[B12] Lee S. P. Sung I. Kim J. H. Lee S. Park H. S. Shim C. S. (2017). Factors impacting patient cooperation during elective gastroscopy. Kor. J. Int. Med. 32 (5), 819–826. 10.3904/kjim.2015.393 28823144 PMC5583449

[B13] Lopes G. S. Bielinski S. Moyer A. M. Jacobson D. J. Wang L. Jiang R. (2021). Sex differences in type and occurrence of adverse reactions to opioid analgesics: a retrospective cohort study. BMJ Open 11 (6), e044157. 10.1136/bmjopen-2020-044157 34193479 PMC8246359

[B14] Loyd D. R. Murphy A. Z. (2009). The role of the periaqueductal gray in the modulation of pain in males and females: are the anatomy and physiology really that different? Neural Plast. 2009, 462879. 10.1155/2009/462879 19197373 PMC2633449

[B15] Lu M. Liu J. Wu X. Zhang Z. (2023). Ciprofol: a novel alternative to propofol in clinical intravenous anesthesia? Biomed. Res. Int. 2023, 7443226. 10.1155/2023/7443226 36714027 PMC9879693

[B16] Ma B. Li Y. Leng C. Ji A. Zhang N. Tao X. (2025). A comparative evaluation of the safety and efficacy of oliceridine and sufentanil in gastrointestinal endoscopy: a single-center, randomized controlled trial. Drug Design Develop. Therapy 19, 5111–5121. 10.2147/DDDT.S512529 40546662 PMC12182078

[B17] Mogil J. S. (2012). Sex differences in pain and pain inhibition: multiple explanations of a controversial phenomenon. Nat. Rev. Neurosci. 13 (12), 859–866. 10.1038/nrn3360 23165262

[B18] Muller P. Y. Milton M. N. (2012). The determination and interpretation of the therapeutic index in drug development. Nat. Rev. Drug Discov. 11 (10), 751–761. 10.1038/nrd3801 22935759

[B19] Nasser S. A. Afify E. A. (2019). Sex differences in pain and opioid mediated antinociception: modulatory role of gonadal hormones. Life Sci. 237, 116926. 10.1016/j.lfs.2019.116926 31614148

[B20] Neilson L. J. Rees C. J. (2025). Patient experience as a quality marker in gastrointestinal endoscopy. Best practice and research. Clin. Gastroenterol. 76, 102029. 10.1016/j.bpg.2025.102029 40610180

[B21] Nguyen N. Pan Z. Smith C. Friedlander J. A. (2024). Transnasal endoscopy ease score “TNEase score” to evaluate patient tolerance of unsedated transnasal endoscopy. J. Pediatr. Gastroenterol. Nutr. 78 (2), 381–385. 10.1002/jpn3.12102 38374574

[B22] Niesters M. Dahan A. Kest B. Zacny J. Stijnen T. Aarts L. (2010). Do sex differences exist in opioid analgesia? A systematic review and meta-analysis of human experimental and clinical studies. Pain 151 (1), 61–68. 10.1016/j.pain.2010.06.012 20692097

[B23] Ortegal G. H. Barbosa E. C. Faria P. C. Couto J. V. Silva G. C. Souza M. H. (2024). Ciprofol *versus* propofol for adult sedation in gastrointestinal endoscopic procedures: a systematic review and meta-analysis. Minerva Anestesiol. 90 (11), 1013–1021. 10.23736/S0375-9393.24.18203-X 39545657

[B24] Pleym H. Spigset O. Kharasch E. D. Dale O. (2003). Gender differences in drug effects: implications for anesthesiologists. Acta Anaesthesiol. Scand. 47 (3), 241–259. 10.1034/j.1399-6576.2003.00036.x 12648189

[B25] Roche D. J. O. King A. C. (2015). Sex differences in acute hormonal and subjective response to naltrexone: the impact of menstrual cycle phase. Psychoneuroendocrinology 52, 59–71. 10.1016/j.psyneuen.2014.10.013 25459893 PMC4482338

[B26] Sorge R. E. Totsch S. K. (2017). Sex differences in pain. J. Neurosci. Res. 95 (6), 1271–1281. 10.1002/jnr.23841 27452349

[B27] Tang Z. Yin G. Yu Y. Luo Y. Tian S. Zhang Q. (2025). Determination of ED(90) and ED(99) of oliceridine combined with propofol in inhibiting responses to gastroscope insertion: a biased coin up-and-down design. BMC Anesthesiol. 25 (1), 175. 10.1186/s12871-025-03052-8 40217139 PMC11987354

[B28] Wolf A. Unterberg M. Witowski A. Adamzik M. Wolf A. (2024). Efficacy, safety, and side effects of oliceridine in acute postoperative pain, a protocol for a systematic review and meta-analysis. PLoS One 19 (2), e0299320. 10.1371/journal.pone.0299320 38422105 PMC10903901

[B29] Zhou J. Wang L. Zhong Z. Yuan L. Huang J. Zou P. (2025). Pharmacological mechanism and clinical application of ciprofol. Front. Pharmacol. 16, 1572112. 10.3389/fphar.2025.1572112 40201700 PMC11975953

