# Supplementary material for: Median effective dose and 95% effective dose of oliceridine combined with ciprofol for painless gastroscopy: a dose-finding study based on Dixon’s up-and-down method
Source: Front Pharmacol. 2026 Sep 4;17:1918438. doi: 10.3389/fphar.2026.1918438 (PMC13585511; doi:10.3389/fphar.2026.1918438)
Supplement: Supplementary file 1 [file Table1.docx]

Supplementary Table 1. Comparison of perioperative hemodynamics and oxygenation status at different times

|  | Male(n=29) | | *P* | Female(n=32) | | *P* |
| --- | --- | --- | --- | --- | --- | --- |
|  | Positive  (n=14) | Negative  (n=15) |  | Positive  (n=14) | Negative  (n=18) |  |
| **MAP(mmHg)** | |  |  |  |  |  |
| T1 | 100.50±7.06 | 101.47±7.41 | 0.722 | 101.14± 10.78 | 98.67±11.90 | 0.548 |
| T2 | 92.57±7.45* | 85.20±8.06*** | 0.017 | 93.57±7.40* | 83.72±8.38*** | 0.002 |
| T3 | 81.64±7.79*** | 84.60±7.36*** | 0.388 | 78.50±6.99*** | 82.17±10.16*** | 0.258 |
| T4 | 91.07±7.40*** | 94.20±8.15** | 0.290 | 89.64±7.14*** | 90.94±7.66** | 0.672 |
| Group Main Effect | F=6530.852; *P*<0.001 | |  | F=4959.832; *P*<0.001 | |  |
| Time Main Effect | F=36.393; *P*<0.001 | |  | F=49.000; *P*<0.001 | |  |
| Group-by-Time Interaction Effect | F=5.598; *P*=0.004 | |  | F=6.638; *P*<0.001 | |  |
| **HR(bmp)** | |  |  |  |  |  |
| T1 | 81.14±11.66 | 82.53±9.78 | 0.766 | 87.64±9.95 | 87.17±10.24 | 0.896 |
| T2 | 75.79±13.84** | 66.40±8.51*** | 0.035 | 81.43±9.22* | 74.50±9.64*** | 0.049 |
| T3 | 71.29±9.66** | 68.73±10.12*** | 0.494 | 71.79±8.92*** | 73.50±9.24*** | 0.601 |
| T4 | 74.86±8.01** | 73.80±7.73*** | 0.673 | 78.71±5.97** | 77.78±7.16*** | 0.696 |
| Group Main Effect | F=1916.712; *P*<0.001 | |  | F=2009.693; *P*<0.001 | |  |
| Time Main Effect | F=53.272; *P*<0.001 | |  | F=15.279; *P*<0.001 | |  |
| Group-by-Time Interaction Effect | F=13.320; *P*<0.001 | |  | F=1.127; *P*=0.337 | |  |
| **SpO_2_ (%)** | |  |  |  |  |  |
| T1 | 97.00(97.00,98.00) | 98.00(97.00,99.00) | 0.398 | 97.00(96.75, 99.00) | 97.00(97.00,98.00) | 0.623 |
| T2 | 99.00(98.00,100.00)*** | 99.00(98.00, 99.00)* | 0.366 | 98.00(98.00,99.00) * | 98.00(98.00, 100.00)** | 0.098 |
| T3 | 98.00(98.00,99.00) | 99.00(98.00, 100.00) | 0.310 | 98.00(98.00,99.00) | 98.00(97.75, 99.00) | 0.152 |
| T4 | 97.00(96.75,98.00) | 98.00(97.00,99.00) | 0.084 | 97.50(97.00, 98.00) | 98.00(97.00, 99.00) | 0.566 |
| Group Main Effect | Waldχ^2^=4.244 P=0.039 | |  | Waldχ^2^=3.231 P=0.045 | |  |
| Time Main Effect | Waldχ^2^=62.010;*P*<0.001 | |  | Waldχ^2^=38.18; *P*<0.001 | |  |
| Group-by-Time Interaction Effect | Waldχ^2^=7.324; *P*=0.062 | |  | Waldχ^2^=7.008; *P*=0.072 | |  |
